# Big Five Traits and Tolerance for Uncertainty as Protective Factors of Subjective Well-Being of Students in Martial Law Conditions

**DOI:** 10.5964/ejop.17767

**Published:** 2026-05-29

**Authors:** Olena V. Zavhorodnia, Mariia V. Shepelova

**Affiliations:** 1 Department of Psychology of Creativity, H. S. Kostiuk Institute of Psychology of the National Academy of Educational Sciences of Ukraine, Kyiv, Ukraine; 2 Department of Psychology of Creativity, H. S. Kostiuk Institute of Psychology of the National Academy of Educational Sciences of Ukraine, Kyiv, Ukraine; 3 Department of Humanitarian Disciplines, National University of Food Technologies, Kyiv, Ukraine; University of Western Australia, Perth, Australia

**Keywords:** emotional stability, openness, novelty, tension, prolonged stress

## Abstract

In the context of prolonged martial law and pervasive uncertainty, the psychological well-being of student populations becomes especially vulnerable. This study examines how personality dispositions — namely, the Big Five traits and tolerance for uncertainty — relate to subjective well-being among Ukrainian university students navigating wartime conditions. Using data from 147 participants (*M*_age_ = 24), predominantly women (78%), studying across various programs in Kyiv, Kharkiv, and Zhytomyr, the research explored direct, mediating, and moderating pathways of influence. The analysis revealed a complex web of interrelations: openness partially mediated the effects of extraversion (*p* = .031), novelty tolerance (*p* = .012), and general uncertainty tolerance (*p* = .008) on subjective well-being. Novelty tolerance also mediated the link between agreeableness and well-being (*p* = .004). Emotional stability emerged as a significant moderator, enabling the protective effects of openness on well-being (*p* = .010), while the protective role of extraversion declined with age (*p* = .018). One particularly noteworthy finding was the unexpected negative association between agreeableness and well-being. In the wartime context, this may reflect a psychological overload among highly agreeable individuals — especially women — facing intense social expectations and emotional caregiving demands, often at the expense of their resources. Future research may benefit from exploring additional mediating variables such as optimism, perceived social support, coping strategies, and cognitive flexibility to better understand adaptive mechanisms under sustained stress.

The relevance of this study is driven by the need for a deeper understanding of the impact of personality dispositions on subjective well-being under martial law conditions. While the influence of personality traits on mental health has been well studied in peacetime (Anglim & Horwood, 2021), their role in prolonged crises remains insufficiently explored.

This study was conducted during the ongoing war in Ukraine, a context of chronic threat, infrastructure disruption, and heightened psychological strain. Unlike short-term emergencies, prolonged crises reshape social environments and limit access to coping resources. In such conditions, stable personality traits may interact with external stressors in unique ways, altering known patterns of vulnerability and resilience.

Understanding which dispositional factors support adaptation under martial law is crucial for developing targeted mental health interventions, improving risk assessment, and strengthening psychological support efforts for war-affected populations.

Existing studies have examined psychological factors of resilience during the pandemic or at the onset of the war; however, the long-term consequences remain underexplored.

Initial expectations for a swift end to the war were higher, whereas prolonged stress may involve different mechanisms of action. Most research focuses on negative consequences, whereas protective factors — such as optimism, tolerance for uncertainty, and social support — remain insufficiently studied. Longitudinal research has begun to address these gaps, showing that personality traits such as extraversion, emotional stability, and conscientiousness are associated with sustained well-being even under crisis conditions (Anglim et al., 2020; Anglim & Horwood, 2021; Winzer et al., 2021). Meta-analytic findings further confirm that subjective well-being develops across the lifespan and is shaped by stable personality dispositions (Buecker et al., 2023). Additionally, tolerance for ambiguity and uncertainty has been identified as a contributing factor to happiness (Zuo, 2024) and is negatively associated with perceived stress and anxiety among students (Zuo, 2025).

## Subjective Well-Being

Subjective well-being is an individual’s satisfaction level with their emotional state, relationships, health, activities, and life. It is characterized by the predominance of positive emotions and mood and serves as a key indicator of psychological well-being (Diener et al., 2003). A high subjective well-being level contributes to psychological resilience, stress adaptability, and overall vitality (Buecker et al., 2023). Conversely, a low level of subjective well-being manifests through the dominance of negative emotions, tension, and mood decline, which can have serious effects on mental health (Carlson, 2024). Research on subjective well-being primarily examines emotional responses, life satisfaction, and individual differences that account for variations in happiness among people (Steel et al., 2008). In this context, particular attention has been given to personality traits, especially those defined by the Big Five model.

## Personality Traits

Personality traits are among the key factors influencing mental health (Jiao et al., 2023). Research confirms that subjective well-being is associated with extraversion, emotional stability, conscientiousness, and agreeableness (Serrano et al., 2020). Studies indicate that neuroticism and extraversion are substantial predictors of subjective well-being, while conscientiousness and agreeableness show a weaker correlation (DeNeve & Cooper, 1998; Steel et al., 2008). Additionally, emotional stability/neuroticism is closely associated with mental health and is considered a potential predictor of subjective well-being (Strohmaier et al., 2024).

Soto (2015) identified a notable correlation between personality traits and aspects of subjective well-being. The study concluded that individuals with higher levels of extraversion, agreeableness, conscientiousness, and emotional stability were more satisfied with life. His research suggested that personality traits and subjective well-being can prospectively predict changes in each other (Soto, 2015). However, there is a lack of longitudinal studies, and sufficient evidence on the impact of subjective well-being on personality traits remains limited.

Cohort and meta-analytic studies (Buecker et al., 2023; Winzer et al., 2021) indicate that subjective well-being is dynamic and can change depending on age, social circumstances, and an individual’s adaptive strategies. For example, during the COVID-19 pandemic, social restrictions reduced the direct influence of extraversion on well-being (Jiao et al., 2023), while a lack of social interaction made extraverts more vulnerable to depression (Strohmaier et al., 2024).

Psychologists have developed various models to classify core personality traits, with the Big Five model being the most widely accepted, although alternative models also exist. Some traits are considered higher-order individual differences (such as extraversion, agreeableness, conscientiousness, openness to experience, and neuroticism in the Big Five model), while many other traits are viewed as lower-order individual differences (Wei et al., 2022).

In psychological research, higher-order traits have received significant attention, whereas lower-order personality traits, such as tolerance for uncertainty, have been studied less extensively. However, examining lower-order constructs with the Big Five traits can provide a deeper understanding of how individual differences (personality) influence subjective well-being. Accordingly, this study aims to enhance the current comprehension of the influence of higher-order personality traits and tolerance for uncertainty on subjective well-being.

## Tolerance for Uncertainty

Tolerance for uncertainty is the ability to perceive ambiguous situations without experiencing significant stress or anxiety (Budner, 1962). This trait is particularly important in conditions of instability and unpredictability, such as armed conflicts, economic crises, or pandemics. Budner (1962) defined tolerance for uncertainty as a personal characteristic that indicates an individual’s inclination to view ambiguous situations as favourable.

Hillen et al. (2017) interpret it as a combination of negative and positive psychological responses — cognitive, emotional, and behavioural — arising from the awareness of not knowing certain aspects of the world. According to Budner, tolerance for uncertainty encompasses cognitive, emotional, and behavioural dimensions, representing an individual’s ability to face situations with insufficient information about the future without experiencing excessive stress or anxiety (Budner, 1962).

Researchers suggest that tolerance for uncertainty is linked to subjective well-being, especially in times of significant instability (Carlson, 2024). In situations where information about the future is limited, and individuals face information warfare and prolonged martial law, a high level of tolerance for uncertainty may aid in maintaining emotional balance and facilitating effective decision-making.

At the same time, some studies underscore potential negative consequences. For example, Zuo (2025) indicates that in conditions of high responsibility, tolerance for uncertainty may elevate stress levels.

Available studies of tolerance for uncertainty highlight its significant impact on emotional state, decision-making capabilities, and subjective well-being. During times of global instability and uncertainty, particularly in wartime, it is crucial to consider this factor in psychological research and in practices aimed at maintaining subjective well-being.

This study aims to determine the role of personality dispositions (Big Five traits and tolerance for uncertainty) in the protection of students’ subjective well-being in wartime uncertainty. The study seeks to establish their interrelations and outline the mediation and moderation effects in the context of the influence of personality dispositions on subjective well-being.

## Hypothesis

Based on previous empirical and theoretical studies (Anglim et al., 2020; Busseri & Erb, 2024; Carleton et al., 2007; Singhal et al., 2021; Soto, 2015; Steel et al., 2008; Zuo, 2025, 2024), the following hypotheses were formulated regarding the relationship between personality traits, tolerance for uncertainty, and subjective well-being among university students during martial law:

**H1.** Higher levels of emotional stability (i.e., lower neuroticism) and extraversion will be negatively associated with students’ subjective well-being decline. These traits have consistently demonstrated positive correlations with well-being indicators across samples and stress-related contexts, including during the COVID-19 pandemic (Anglim et al., 2020; Soto, 2015; Steel et al., 2008; Winzer et al., 2021).
**H2.** Conscientiousness will show a moderate negative association with a decline in subjective well-being under conditions of prolonged uncertainty and threat. This trait is linked to adaptive behaviour, self-regulation, and the capacity for long-term planning, all of which foster resilience in times of crisis (Anglim et al., 2020; Singhal et al., 2021; Soto, 2015; Steel et al., 2008).
**H3.** Agreeableness and openness to experience are expected to demonstrate weak to moderate negative associations with a decline in subjective well-being. Although empirical findings for these traits are less consistent, cross-cultural studies — including research conducted in India — report predominantly positive, albeit variable, associations (Busseri & Erb, 2024; Singhal et al., 2021; Tanksale, 2015). Meta-analytical reviews also highlight their adaptive potential under conditions of social stress (Anglim et al., 2020; Busseri & Erb, 2024; Singhal et al., 2021).
**H4.** Tolerance for uncertainty — specifically the readiness to engage with novelty and ambiguity — is expected to negatively relate to a decline in subjective well-being. Research suggests that, in chronically uncertain environments, the ability to tolerate ambiguity reduces anxiety and helps preserve psychological well-being (Carleton et al., 2007; Zuo, 2024, 2025).

## Method

### Participants

The study included both men and women (a total of 147 participants) aged 17 to 49, primarily students from the cities of Kyiv, Kharkiv, and Zhytomyr, Ukraine. The sample comprised 78% women and 22% men. The age range for women was from 17 to 45 years, while for men, it extended from 17 to 49 years. The sample included 59 students (40%) enrolled in full-time programs and 88 individuals (60%) pursuing part-time studies. Full-time students were predominantly aged 17–22, while part-time participants ranged from 18 to 49 years. The sample was randomly selected and did not require the presence of any specific mental disorders. The research was conducted in 2024 under martial law during the full-scale Russian invasion. The participants’ average age was 24 years (*M* = 24.09, *SD* = 7.6).

### Procedure

The organisation of the study involved providing standardized questionnaires through a Google Form. Respondents received the complete set of assessment methods and instructions. Participation in the study was voluntary and anonymous, with informed consent obtained from each participant. After processing the results, each participant received a detailed interpretation of their outcomes and had the opportunity to seek additional support if needed. H. S. Kostiuk Institute of Psychology of the National Academy of Educational Sciences of Ukraine Research Ethics Committee approved the study protocol and materials, Extract from Minutes No. 2/7 dated December 18, 2023.

### Measures

#### Subjective Well-Being

The Six-Cluster Ukrainian language version of the Subjective Well-Being Scale (Perrudet-Badoux et al., 1988) was used as the primary assessment tool. This scale serves as a tool for evaluating participant subjective well-being, particularly its emotional component, across six clusters. The inventory comprises 17 statements/questions that assess the degree of positive/negative emotions and satisfaction with mood, health, social environment, and daily activities. Low scores on this scale indicate high subjective well-being, characterised by the predominance of positive emotions, good mood, and satisfaction with aspects such as health, daily activities, and social environment. Conversely, high scores reflect a low level of subjective well-being, including negative emotions, mood deterioration, and dissatisfaction with various aspects of life.

Due to the reversed nature of several indicators, cluster labels were refined to enhance interpretability; however, these semantic adjustments did not alter the scale’s internal structure or scoring algorithm. The revised cluster scales and their respective Cronbach’s alpha values in the current sample were as follows: Tension (α = .325), Symptoms of Major Psycho-Emotional Issues (α = .646), Mood Deterioration (α = .435), Social Environment Significance (higher scores reflect lack of social support and loneliness; α = .508), Self-Assessment of Health Status (higher scores indicate dissatisfaction with personal health; α = .816), Satisfaction with Daily Activities (higher scores indicate lower satisfaction; α = .449), and Overall Subjective Well-Being Decrease (α = .860). Each item was rated on a 7-point Likert scale and included both directly and reverse-worded statements.

The Perrudet-Badoux et al. (1988) scale has a long history of application in Ukrainian-language psychological research, particularly in studies examining the emotional and cognitive components of subjective well-being. For example, Kryazh and Levenets (2023) used the instrument to assess emotional discomfort in a comparative analysis of personality openness profiles. Similarly, Himayeva and Malofeikina (2019) employed it to explore associations between subjective well-being and personal maturity levels among students in different educational formats. More recently, studies such as Zavhorodnia et al. (2025) have used the scale to compare subjective well-being data collected in 2024 against pre-invasion baselines established prior to Russia’s full-scale escalation in 2022.

#### “Big Five” Personality Traits

This study employed the Ukrainian adaptation (TIPI-UKR) of the brief personality inventory known as the 10-Item Big Five Inventory (BFI-10; Rammstedt & John, 2007), developed and psychometrically validated by Klimanska and Haletska (2019). The adaptation followed established procedures including forward and backward translation, descriptor refinement, and evaluation of internal consistency, test–retest reliability, and construct validity.

In the present study, Cronbach’s alpha coefficients for the TIPI-UKR subscales were as follows: Extraversion (α = .483), Agreeableness (α = .477), Conscientiousness (α = .529), Emotional Stability (α = .596), and Openness to Experience (α = .737). These values are consistent with prior validation and support the acceptable internal consistency of the instrument for use under time-limited conditions and elevated emotional strain typical of crisis or wartime contexts.

The scale comprises 10 items representing key personality traits, each rated on a 7-point Likert scale, with both positively and negatively worded statements.

#### Tolerance for Uncertainty

An adapted Ukrainian-language version of the Intolerance of Ambiguity Scale, originally proposed by Budner (1962), was used in this study. The adaptation was developed and psychometrically validated by Barko and Ostapovych (2019), demonstrating acceptable reliability (Cronbach’s α = .65–.69) and offering standardized norms for Ukrainian samples.

The instrument comprises 16 items evaluating attitudes toward ambiguous situations, rated on a 7-point Likert scale. Both directly worded and reverse-coded items are included. In the present study, internal consistency coefficients were notably low across subscales: Tolerance for Novelty (α = .247), Tolerance for Complexity (α = .262), Tolerance for Insolubility (α = –.135), and General Tolerance for Uncertainty (α = .270).

These diminished alpha values may reflect elevated psycho-emotional strain among respondents under martial law conditions, which could have disrupted response coherence on cognitively demanding subscales involving abstract or ambiguous scenarios.

### Statistical Analysis

The analysis employed descriptive statistics, Pearson’s correlation analysis, and conditional process modelling (Hayes, 2022), including mediation (Model 4) and moderation (Model 1) analyses with bias-corrected bootstrap confidence intervals (5,000 resamples), to examine indirect and interaction effects of personality dispositions on subjective well-being. The analysis was conducted using SPSS Statistics 27.0.1. package.

Open data, materials in original and English versions, and commands to recreate the analyses for the article can be found on the Open Science Framework at Shepelova (2025).

## Results

### Preliminary Analyses, Descriptive Statistics and Intercorrelations

As an initial step in the authors’ study, fundamental statistical procedures were conducted to assess the feasibility of further statistical analysis of the empirical data. Table 1 presents the values for means, standard deviations, skewness, and kurtosis. Skewness and kurtosis were measured to assess the suitability of parametric analyses. The data were considered normally distributed as skewness and kurtosis values were below 1.0.

**Table 1:** Means, Standard Deviations, Skewness and Kurtosis of the Variables

Variable	*M*	*SD*	*Skewness*	*Kurtosis*
Subjective well-being				
Tension	12.32	3.44	-.01	.02
Symptoms Associated with Psycho-Emotional Distress	14.99	4.77	.10	-.41
Mood Deterioration	5.98	2.38	.79	.61
Significance of Social Environment	7.95	3.09	.46	-.41
Dissatisfaction with Health Status	7.46	2.88	.22	-.52
Dissatisfaction with Daily Activities	10.69	3.51	.27	-.38
General Decline in Subjective Well-Being	59.39	15.41	.22	.25
Big Five Traits				
Extraversion	9.29	2.31	-.34	-.33
Agreeableness	8.84	2.13	-.31	-.14
Conscientiousness	10.33	2.15	-.56	-.15
Emotional stability	7.83	2.70	.03	-.64
Openness	10.46	2.38	-.82	.85
Tolerance for uncertainty				
Tolerance for novelty	16.41	3.60	.23	-.49
Tolerance for complexity	40.59	4.87	-.11	-.12
Tolerance for insolubility	13.36	2.37	.13	-.37
Tolerance for uncertainty	70.36	6.84	.24	.40

The results of Pearson’s correlation analysis (Table 2) indicate that subjective well-being and its components exhibit significant associations with Big Five personality traits and tolerance for novelty.

**Table 2:** Correlations Between Components of Subjective Well-Being With Big Five Traits and Tolerance to Uncertainty

Variable	Т	SP	DM	SЕ	SH	SА	DSW
Extraversion	-.11	-.21**	-.31**	-.27**	-.20*	-.18*	-.27**
Agreeableness	.15	.07	.12	.15	.10	.16*	.16*
Conscientiousness	-.10	-.18*	-.13	-.13	-.12	-.29**	-.21**
Emotional stability	-.41**	-.59**	-.35**	-.16*	-.34**	-.29**	-.49**
Openness	-.26**	-.16*	-.36**	-.16*	-.20**	-.16*	-.27**
Tolerance to novelty	-.22**	-.08	-.10	-.11	-.15	-.10	-.16*
Tolerance to complexity	-.04	-.01	-.15	.02	.00	.07	-.02
Tolerance to insolubility	.01	.01	-.16*	-.10	.07	.00	-.05
General tolerance to uncertainty	-.14	-.05	-.22**	-.08	.10	.01	-.12

*Note.* T – Tension, SP – Symptoms Associated with Psycho-Emotional Distress (sleep disturbances, increased anxiety, excessively sharp reactions to certain events), DM – Mood Deterioration, SE – Significance of Social Environment (problems in the social environment, lack of social support, feelings of loneliness, etc.), DSH – Dissatisfaction with Health Status, DSA – Dissatisfaction with Daily Activities, DSW – General Decline in Subjective Well-Being.

* *p* < .05. ** *p* < .01.

A correlation analysis of the relationships between personality dispositions was also conducted (Table 3).

**Table 3:** Correlations Between Big Five traits, Dispositional Optimism, and Tolerance for Uncertainty

Variable	1	2	3	4	5	6	7	8	9
1. Tolerance to novelty	–	.09	-.03	.58**	.14	.43**	.02	.04	.40**
2. Tolerance to complexity		–	.08	.79**	.17*	.06	.04	.08	.18*
3. Tolerance to insolubility			–	.39	-.02	.03	-.05	.06	.14
4. Tolerance to uncertainty				–	.18*	.28**	.03	.10	.38**
5. Extraversion					–	-.05	.11	-.02	.31**
6. Agreeableness						–	.01	-.07	.07
7. Conscientiousness							–	.06	.10
8. Emotional stability								–	.30**
9. Openness									–

* *p* < .050. ** *p* < .010.

**Figure 1:**
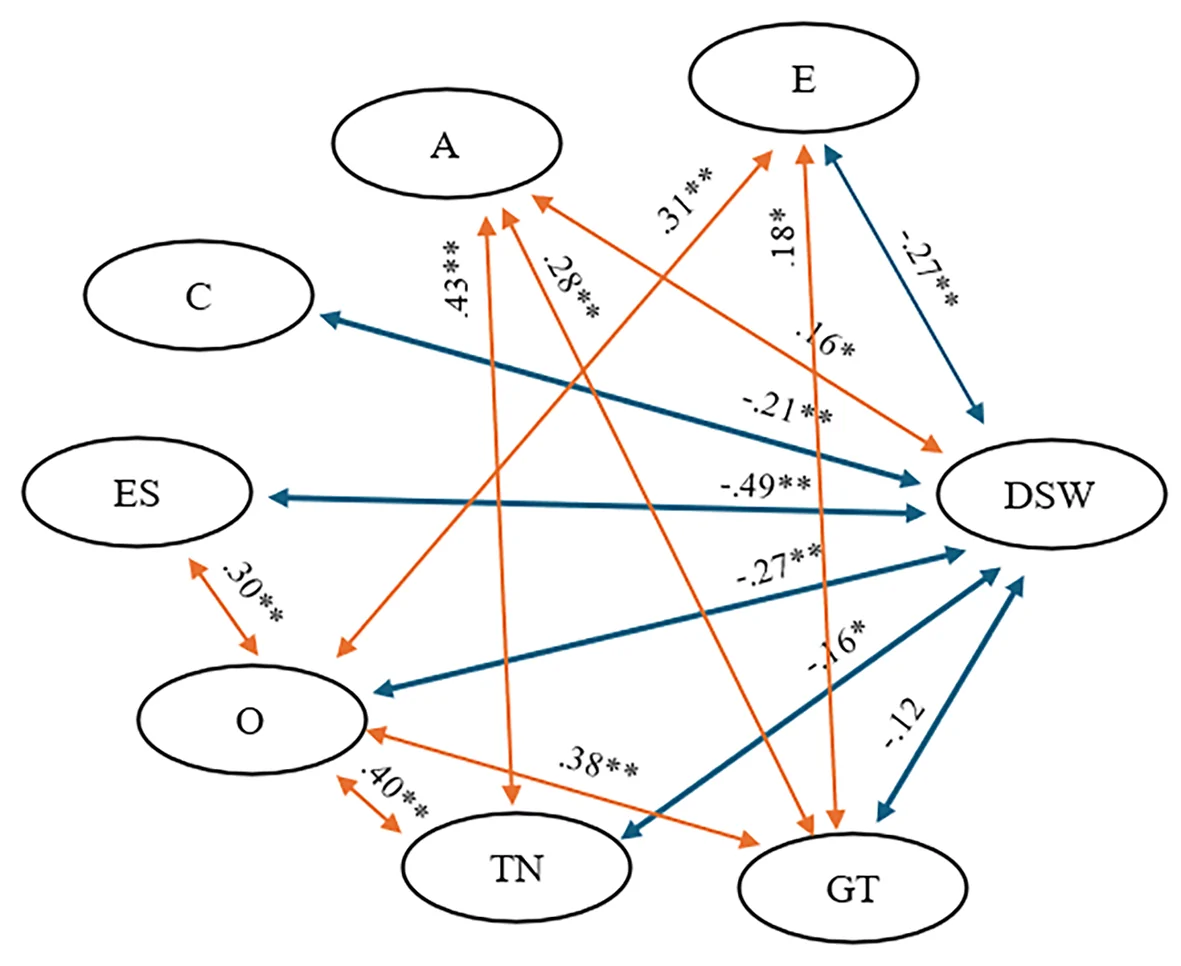
Correlation Model Illustrating Significant Associations Between Personality Traits and Subjective Well-Being Decline *Note.* Blue arrows represent negative correlations, red arrows represent positive correlations. DSW – General Decline in Subjective Well-Being; E – Extraversion, A – Agreeableness; C – Conscientiousness; ES - Emotional stability; O – Openness; TN - Tolerance to novelty; TG – Tolerance to uncertainty. * *p* < .05. ** *p* < .01.

The pattern of significant associations identified in the correlation analysis is further visualized in Figure 1, highlighting the key links between personality traits and subjective well-being decline.

### Mediation and Moderation Analyses

To determine the nature of the relationship between personality dispositions (Big Five traits, tolerance for uncertainty) and subjective well-being decline, as well as to identify mediation and moderation effects, a mediation analysis (Table 4) and a moderation analysis were performed, employing conditional process analysis (Hayes, 2022). Both analyses incorporated bias-corrected bootstrap confidence intervals (5,000 resamples) to ensure robust estimation of indirect effects and interaction terms, minimising reliance on normality assumptions.

**Table 4:** Mediation Analysis Results

Effect	Variable	Β	*p*
E → O → DSW			
Indirect effect	E → O → DSW	-.065	.031
Component effect	E → O	.312	.001
	O → DSW	-.209	.011
Direct effect	E → DSW	-.203	.013
Total Effect	E → DSW	-.269	< .001
A → TN → DSW			
Indirect effect	A → TN → DSW	-.122	.004
Component effect	A → TN	.429	< .001
	TN → DSW	-.284	.001
Direct effect	А → DSW	.282	.001
Total Effect	А → DSW	.160	.001
GT → O → DSW			
Indirect effect	GT → O → DSW	-.102	.008
Component effect	GT → O	.382	< .001
	O → DSW	-.267	.002
Direct effect	GT → DSW	.015	.860
Total Effect	GT → DSW	-.117	.154
TN → O → DSW			
Indirect effect	TN → O → DSW	-.100	.012
Component effect	TN → O	.403	< .001
	O → DSW	-.247	.004
Direct effect	TN → DSW	-.063	.462
Total Effect	TN → DSW	-.163	.046

*Note.* E – Extraversion, A – Agreeableness, O – Openness, TN – Tolerance for Novelty, GT – General Tolerance for Uncertainty; DSW – General Decline in Subjective Well-Being.

Mediation analyses supported several significant indirect pathways, as summarized in Table 4. Specifically, openness was found to partially mediate the effects of extraversion and tolerance for novelty on subjective well-being, while also fully mediating the association between tolerance for uncertainty and well-being.

**Figure 2:**
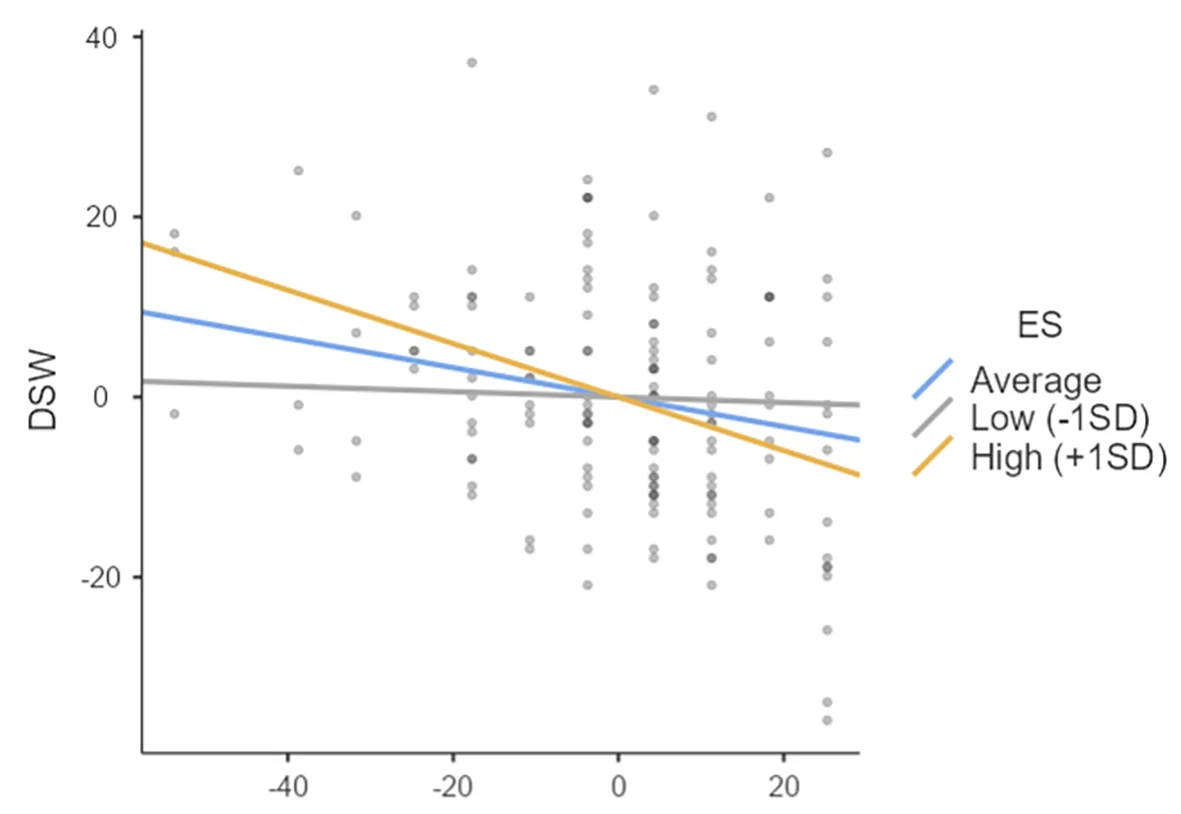
Simple Slopes Plot: Openness as a Predictor of Subjective Well-Being at Varying Levels of Emotional Stability

To examine whether emotional stability (ES) moderates the association between openness (O) and decline in subjective well-being (DSW), a moderation model was tested. A significant interaction effect was found between openness and emotional stability (β = –.007, *p* = .010). Simple slopes analysis revealed that openness predicted higher subjective well-being at high (β = –.297, *p* < .001) and average (β = –.163, *p* = .004) levels of emotional stability, but not at low levels (β = –.475, *p* = .635) (Figure 2).

These results suggest that openness protects against decline in subjective well-being only when emotional stability is average or above. At low levels of emotional stability, openness does not exert a protective effect (*p* = .635).

**Figure 3:**
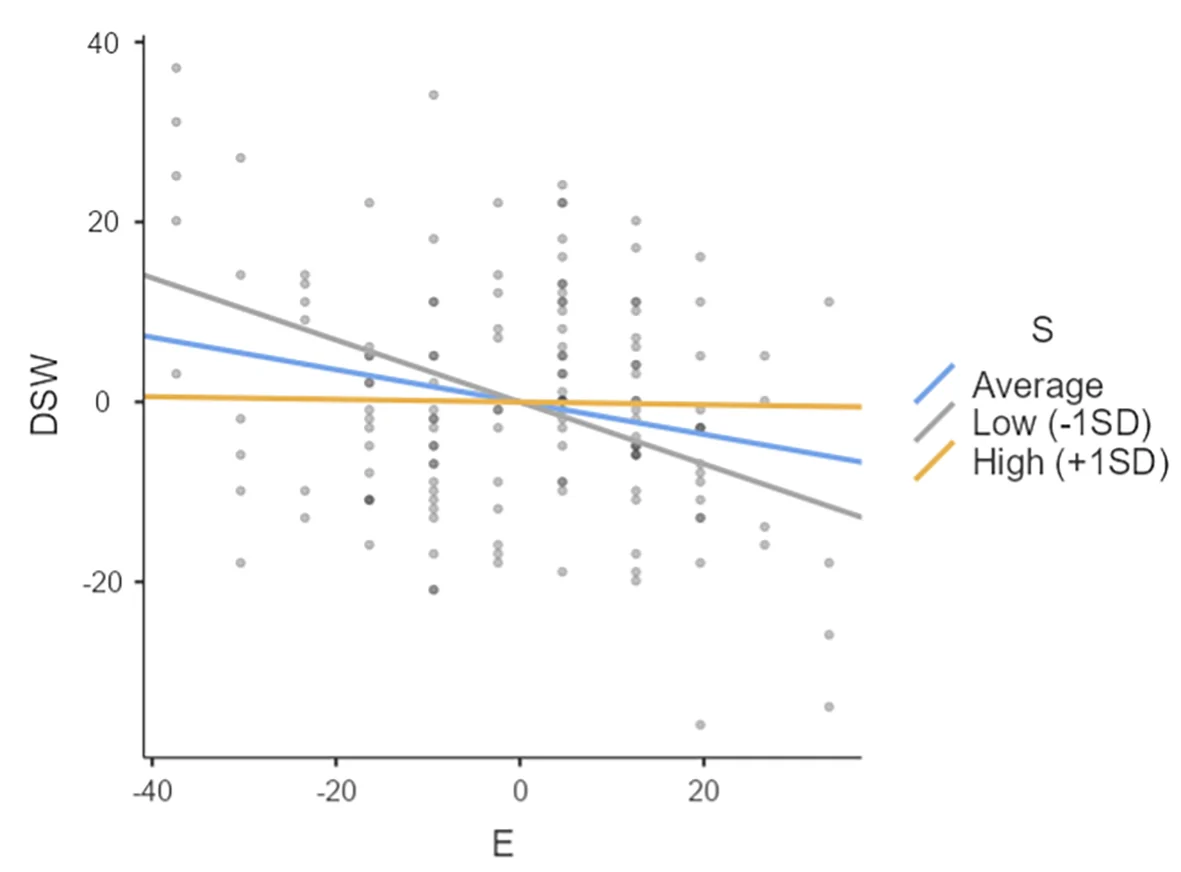
Simple Slopes Plot: Extraversion as a Predictor of Subjective Well-Being at Different Age Levels

To account for age as a covariate, an additional moderation analysis was conducted to examine whether age influenced the relationship between personality traits and decline in subjective well-being (DSW). Extraversion emerged as the only trait for which age acted as a statistically significant moderator. A significant interaction was found between Age (S) and Extraversion (E) (β = .022, *p* = .018), indicating a moderation effect. Simple slopes analysis revealed that extraversion was a strong protective factor among younger participants (β = –.345, *p* < .001), remained significant but weaker at average age (β = –.180, *p* = .005), and was non-significant among older individuals (β = –.015, *p* = .890) (Figure 3).

These results suggest that extraversion functions as a protective factor against decline in subjective well-being predominantly for younger individuals, with its buffering effect diminishing with age. No significant moderation effects of age were observed for other personality traits or tolerance for uncertainty.

## Discussion

Taken together, the findings provide empirical support for all four hypotheses, albeit with varying degrees of consistency.

**H1** was confirmed. Emotional stability and extraversion emerged as statistically significant predictors of subjective well-being. Specifically, emotional stability demonstrated the strongest negative association with a decline in well-being (*r* = –.49, *p* < .01), indicating its leading protective role. Extraversion also showed a significant association (*r* = –.27, *p* < .01), although to a lesser extent.
**H2** was confirmed. Conscientiousness showed a moderate, statistically significant negative association with a decline in well-being (*r* = –.21, *p* < .01), consistent with the hypothesis regarding its adaptive function under stress.
**H3** was partially confirmed. In the case of openness to experience, a stronger-than-expected negative association with a decline in well-being was observed (*r* = –.27, *p* < .01), which contradicts the initial assumption of a weak effect, yet overall supports its contribution. In contrast, agreeableness showed a weak but positive association with indicators of a decline in well-being (*r* = .16, *p* < .05), contrary to our expectation regarding its protective function. This may indicate a potential paradoxical effect in the context of war, where heightened sensitivity to social expectations and the needs of others may reduce subjective well-being.
**H4** was partially confirmed. Only tolerance for novelty demonstrated a statistically significant negative association with a decline in well-being (*r* = –.16, *p* < .05), partially supporting the hypothesis regarding its protective role. Other aspects of uncertainty tolerance — specifically tolerance for complexity, ambiguity, and the overall index — did not show statistically significant associations with subjective well-being.

Importantly, the mediation analysis revealed several indirect pathways: openness to experience partially mediated the effects of both extraversion and tolerance for novelty on subjective well-being and fully mediated the relationship between general tolerance for uncertainty and well-being. These findings suggest that openness may serve as a psychological resource in the context of adapting to novel or ambiguous conditions.

The moderation analysis indicated that emotional stability alters the strength of the association between openness and subjective well-being. Specifically, openness was positively associated with well-being only among individuals with moderate or high levels of emotional stability; under conditions of low stability, its protective effect was not observed.

An additional moderation analysis examining the role of age confirmed that extraversion exerted a robust protective effect among younger participants, which gradually diminished with age. No significant age-based moderation effects were found for other personality traits or dimensions of uncertainty tolerance.

### Extraversion and Subjective Well-Being

The authors’ study shows that extraversion directly correlates with well-being, which partially aligns with previous findings by Diener and Lucas (2008), who emphasize its link to positive affect. A meta-analysis by Steel et al. (2008) also confirmed the significant influence of extraversion.

The authors’ results indicate that extraversion is not a primary predictor of well-being, as its correlation with well-being is less significant than that of emotional stability. Contextual factors, such as the impact of personality traits on well-being under crisis conditions, may explain this. Extraversion may become less significant in times of war and instability, while emotional stability becomes critically important (Hudek-Knežević & Kardum, 2009). Additionally, Wilt and Revelle (2009) note that extraverts may be more emotionally sensitive to changes, which can reduce their resilience in stressful situations and potentially offset the positive influence of extraversion on well-being.

Research suggests that extraversion may not directly predict well-being. Instead, its impact may be mediated through several mechanisms. First, through social activity (Diener & Lucas, 2000), individuals with a high number of social interactions tend to experience improved mood. Second, through positive affect: extraverts are more likely to experience frequent and intense positive emotions, which contribute to higher levels of well-being (Diener et al., 2003). Third, through social support: extraverts are more likely to build and maintain supportive relationships, which serve as a buffer against stress and enhance overall well-being (Soto, 2015). However, when social interactions are restricted (e.g., due to war or a pandemic), extraversion loses its expected advantage. Strohmaier et al. (2024) observed that extraverts could experience increased susceptibility to depression because they rely on social interaction. This is also supported by the results of Anglim and Horwood (2021) found that social restrictions during the COVID-19 pandemic reduced the direct extraversion influence on well-being.

The authors examined whether extraversion directly and indirectly influences decline in subjective well-being (DSW) through openness to experience. The model presented in Table 4 indicated partial mediation, suggesting that openness partly explains the relationship between extraversion and DSW. This finding is partly consistent with Diener and Lucas’s (2008) proposition that extraverted individuals tend to be more open to new experiences, which may enhance their well-being.

However, the mediation effect appeared limited, likely due to the crisis context of the study. Under such conditions, emotional stability plays a more pronounced protective role than extraversion or openness. Moreover, extraversion does not always manifest as behavioural openness; extraverts may be socially active primarily within familiar environments and not necessarily inclined toward seeking novel experiences.

### Openness and Well-Being

Openness directly correlates with well-being, although its connection was found to be weaker than the relationship between well-being and emotional stability. The authors’ results contradict Diener and Lucas (2008), who, in review studies, found no strong link between openness and well-being. Meanwhile, the meta-analysis by Steel et al. (2008) confirmed a significant, although relatively moderate, contribution of openness to well-being.

In the structural model by Romero et al. (2009), which considers emotional stability, extraversion, agreeableness, and conscientiousness as predictors of well-being, openness plays no significant role. In this model, openness was not considered a key predictor of well-being, highlighting the ambiguity of its role. This emphasizes the contextual dependency of its influence and the need to account for other personality resources.

This suggests that the direct impact of openness is realized only in specific contexts and under favourable personality or situational factors.

Thus, openness helps reduce tension and adapt to uncertainty, aligning with contemporary research on cognitive flexibility. However, the hypotheses regarding the mediating role of tolerance for novelty or uncertainty were not confirmed. Openness has the potential to enhance well-being, but its direct effects are realized only in combination with other factors, particularly emotional stability.

The authors' results contradict those of Lucas and Diener (2008), who found no strong connection between openness and well-being. It can be hypothesized that high openness may even increase cognitive load and stress, which helps explain the discrepancies in the literature.

However, several studies confirm the potentially beneficial role of openness, but only under the condition of favourable moderators. For example, Soto (2015) suggested that high openness enhances well-being, but only in individuals with high emotional stability. That is, openness can serve as a resource for personal growth if the individual possesses sufficient emotional regulation mechanisms.

Thus, high openness may serve as a resource for personal growth, but only in the case of sufficient emotional regulation. Youfi and Brigui (2024) demonstrated that openness is associated with intercultural communication, which may facilitate better adaptation to uncertainty.

Recent research deepens the understanding of the mechanisms through which openness may influence well-being. Fiori et al. (2022) demonstrated that creative individuals adapt more effectively to life changes and experience higher life satisfaction during periods of instability.

Men with higher creativity, as measured by the openness personality factor, live longer: one standard deviation increase in creativity reduces the mortality risk by 12% (Turiano et al., 2012).

It is possible that, in complex or crises situations, individuals with high openness are better at solving challenging tasks and more quickly find new ways to overcome problems.

Given the increasing uncertainty in the world, the authors hypothesized that openness might enhance well-being by fostering tolerance for novelty or uncertainty. However, the authors’ mediation hypotheses were not confirmed.

Therefore, openness has the potential to promote well-being primarily as a factor of cognitive flexibility and adaptation to change (Turiano et al., 2012). However, its positive impact is realized only in combination with emotional stability. Without emotional regulation resources, openness may lead to cognitive overload and stress, which could explain the contradictory findings in the literature.

### Emotional Stability as a Key Predictor of Well-Being

The study conducted by the authors substantiates that emotional stability serves as one of the most significant predictors of well-being, corroborating the results reported by Diener and Lucas (2008) and Diener et al. (2003), who emphasized that emotional stability is more strongly associated with the absence of negative affect than extraversion is with positive affect.

However, the evidence is not entirely consistent. Strohmaier et al. (2024) argue that even a high level of emotional stability does not necessarily ensure well-being, as it may reduce emotional sensitivity.

Thus, while emotional stability helps regulate negative emotions, reduce stress, and prevent catastrophizing, it may also dampen emotional sensitivity and limit intense positive experiences. Carlson (2024) highlights that in unstable situations, emotional stability may be less important than adaptability to uncertainty.

The authors’ findings suggest that the crisis context (war, instability) shifts the importance from extraversion to emotional stability as the key protective factor.

Furthermore, emotional stability moderates the effect of openness on well-being: when emotional stability is high, openness has a direct influence on well-being; however, when emotional stability is low, this effect is no longer present.

This aligns with research by Soto (2015) and DeYoung et al. (2007), which demonstrated that emotional stability acts as a buffer against negative experiences, enabling open individuals to better adapt. Similarly, Hudek-Knežević and Kardum (2009) demonstrated that, under stress, emotional stability becomes the most critical protective factor, while extraversion and openness lose their significance.

### Conscientiousness and Well-Being

The proposed study showed that conscientiousness reduces psycho-emotional symptoms, enhances satisfaction with activities, and is generally directly associated with well-being. There is evidence supporting the authors’ results in other studies. Steel et al. (2008) showed that conscientious individuals are more likely to use planning strategies. Hudek-Knežević and Kardum (2009) found that conscientiousness promotes better stress adaptation through discipline and control over situations. Soto (2015) showed that conscientiousness is associated with long-term improvements in mental health.

The authors’ results are consistent with the “positive personality” concept and research on the impact of a set of traits characteristic of a “positive personality”, particularly conscientiousness, on well-being during the COVID-19 pandemic (Jiao et al., 2023). DeYoung et al. (2007) suggested that conscientiousness may directly enhance well-being through self-discipline and organization.

### Agreeableness and Well-Being

So, in the authors’ study, extraversion, conscientiousness and openness function, albeit to varying degrees, as protectors of well-being. At the same time, agreeableness is not a protector of subjective well-being, but a risk factor: an unexpected weak correlation was found between agreeableness and decreased well-being (*r* = -0,16, *p* < .05). This contradicts traditional views suggesting that agreeableness is associated with better social relationships and emotional well-being (Robins et al., 2002).

However, a meta-analysis of studies where agreeableness is typically associated with higher well-being has shown that the effects depend on context (DeNeve & Cooper, 1998). According to Ashton and Lee (2007), agreeableness can facilitate social integration, but it may also increase the risk of stress due to excessive social involvement.

Possible explanations may include several factors. First, social sensitivity: agreeable individuals may become overly involved in others’ problems, leading to emotional exhaustion. Second, vulnerability to conflict: avoiding confrontation to maintain harmony can cause internal tension (Graziano et al., 2007). Third, dependence on mutual support: agreeableness promotes well-being particularly in the presence of stable social support (Taylor, 2011). In unstable conditions, such support may be insufficient, increasing emotional costs associated with maintaining relationships (Margolis & Lyubomirsky, 2018).

Interestingly, the relationship between agreeableness and well-being is modified by novelty tolerance. In the authors’ study, it was found that with high novelty tolerance, the negative effect of agreeableness on well-being is amplified. This aligns with the suggestion that agreeable individuals with high openness to new experiences may become overly involved in new social situations that require adaptation and significant emotional resources (Hillen et al., 2017). The combination of social empathy and social curiosity may increase the risk of overload, loss of personal boundaries, and cognitive and emotional fatigue (Cohen & Wills, 1985; Kashdan & Steger, 2007). Thus, high social openness may, in certain contexts, work against well-being.

Furthermore, the role of agreeableness changes in conditions of social instability, particularly during crises. In such circumstances, it may manifest not as classic “social pleasantness” but perhaps as communicative flexibility in new, uncertain environments. That is, the functions of this personality trait may change depending on the context. Agreeableness, combined with high novelty tolerance, can both promote adaptation to new situations and increase the risk of emotional exhaustion due to excessive social engagement (Xu & Tracey, 2014).

In chronically stressful contexts, individuals high in agreeableness — especially women — may be more susceptible to emotional exhaustion. This vulnerability stems from hypersocial adaptation: a tendency to prioritize others’ needs, heightened empathy, and altruistic orientation, often at the expense of personal resources. Purvanova and Muros (2010) found women to be significantly more prone than men to emotional exhaustion in demanding environments, consistent with gender role theory. Ariza Toledano and Ruiz-Olivares (2023) similarly reported that prosocial traits like agreeableness and empathy predict burnout among women in helping professions under social turbulence. Wagaman et al. (2015) further identified empathy as a risk factor for secondary traumatic stress through prolonged exposure to others’ suffering.

Our sample, composed predominantly of young female respondents, may have accentuated this dynamic. Accordingly, agreeableness in the context of war may function as a paradoxical risk factor: when social expectations demand constant caregiving, the psychological toll may include fatigue, diminished well-being, and heightened emotional vulnerability.

Therefore, the unexpectedly negative relationship between agreeableness and well-being may be explained by high social sensitivity, emotional strain, and contextual factors. At the same time, in crisis conditions, this trait takes on adaptive functions, which warrants further investigation. The authors’ results highlight the importance of considering the interaction between personality traits and situational context when analysing their impact on subjective well-being.

### Tolerance for Uncertainty and Well-Being

The obtained results indicate that tolerance for novelty and general tolerance for uncertainty influence subjective well-being through the mediating variable — openness. This aligns with theoretical approaches to studying personality adaptation resources and highlights the role of psychological flexibility in maintaining well-being.

Additionally, these findings are consistent with research demonstrating that the ability to accept uncertainty reduces anxiety and enhances stress resilience (Carleton et al., 2007). This is further supported by Xue (2024), who states that tolerance for uncertainty facilitates adaptation through cognitive mechanisms of situation appraisal.

The authors’ results contradict Ashton and Lee (2007), who suggest that tolerance for uncertainty is merely a component of emotional stability rather than an independent factor of well-being. Additionally, Zuo (2024) notes that under high responsibility, tolerance for uncertainty may increase stress. However, the authors’ findings confirm that this construct can function as an adaptive factor.

The author’s data also confirmed that openness mediates the relationship between tolerance for uncertainty and well-being. Tolerance for uncertainty enhances openness, and openness, in turn, fosters subjective well-being. This supports the hypothesis that the ability to accept uncertainty and remain open to various possibilities contributes to better adaptation and psychological comfort.

Openness serves as a protective factor: individuals who are more open to new experiences adapt more effectively to change, integrate new experiences more efficiently, and consequently feel less psychologically vulnerable.

The authors’ results indicate the need to reconsider the impact of tolerance for uncertainty in well-being models and to further explore its influence on adaptability in unstable conditions.

This study offers several strengths that enhance its scientific relevance and potential for future exploration. A comprehensive approach was adopted to examine subjective well-being, addressing both its positive and negative components. This enabled the identification of factors contributing to both the enhancement and deterioration of well-being. The analysis incorporated a wide range of psychological variables, including Big Five personality traits and tolerance for uncertainty and its components — tolerance for novelty, complexity and insolubility, allowing for a nuanced understanding of their roles. The study also holds contextual value, being conducted during martial law in Ukraine, thus providing unique insights into personality functioning under extreme social conditions. Additionally, the use of mediation analysis revealed both direct and indirect pathways of influence, offering avenues for further research.

Despite its contributions, several limitations warrant consideration. The use of convenience sampling resulted in demographic imbalances, notably a predominance of young women from large Ukrainian cities, which restricts the generalizability of the findings to broader socio-demographic groups. The relatively small sample size limited the statistical power of the study, increasing the risk of undetected weak effects (Type II error) and constraining the robustness of conclusions. While the nominal age range was broad (17–49 years), most participants (over 80%) were students under 27, although the inclusion of older individuals may have contributed to variability in certain indicators.

The cross-sectional design does not permit causal inference or assessment of temporal dynamics. Some subscales — particularly within the tolerance for uncertainty questionnaire — exhibited insufficient reliability, necessitating caution in interpreting specific associations. Additionally, key variables such as optimism were not assessed, and the focus was limited to the emotional facet of subjective well-being, omitting its cognitive and behavioural dimensions. Several statistically significant correlations were weak, underscoring the complexity of subjective well-being and the need for more integrative models. Future research with larger, more diverse samples and expanded variable sets will be essential for testing and extending the current findings.

## Conclusion

This study aimed to explore how personality traits, particularly the Big Five factors and tolerance for uncertainty, relate to students’ subjective well-being under martial law conditions in Ukraine. Despite its limitations, the findings illuminate key psychological resources that may facilitate students’ adaptation to prolonged stress and unpredictability.

Traits such as emotional stability, openness to experience, extraversion, and conscientiousness emerged as potential protective factors. Emotional stability played a particularly central role, showing the strongest direct association with well-being. Openness was linked to reduced emotional strain, with part of its effect mediated by emotional stability. Conscientiousness was associated with lower levels of psycho-emotional symptoms and greater satisfaction with activities. Tolerance for novelty, a subcomponent of the broader tolerance for uncertainty construct, also showed positive influence on well-being, partly through its linkage to openness. Both tolerance for novelty and general tolerance for uncertainty exert a protective effect on subjective well-being through the mediation of openness to experience. These results point to a complex interplay of individual traits operating through mediational pathways.

A moderation effect of age was also observed: the protective role of extraversion was most pronounced among younger students but diminished among older participants. This suggests that the adaptive value of personality traits may differ across age groups — a nuance worth considering in psychoeducational and intervention design.

The unexpected negative association between agreeableness and well-being likely reflects the crisis-specific context, where excessive orientation toward interpersonal harmony may heighten emotional strain. This may be especially relevant for women, given the gender-skewed sample comprised mainly of young women from urban Ukrainian settings. Heightened sensitivity to social expectations and the drive to maintain relational stability in times of threat may intensify emotional vulnerability. At the same time, tolerance for novelty appeared to buffer this effect, highlighting the potential for complex moderating dynamics between personality and context.

Although the findings shed light on new aspects of the relationship between personality traits and students’ subjective well-being in the wartime context, they should be regarded as exploratory, given the limited sample size, which is dominated by young women, the mixed age composition, and the reduced reliability of certain subscales. Future studies using larger and more diverse samples, longitudinal approaches, and expanded constructs — such as optimism, social support, meaning in life, and cognitive flexibility — will help refine and extend these insights into youth adaptation under sustained uncertainty.

## Supplementary Materials

**Table:** 

Type of supplementary material	Availability/Access
Data	
Materials	Shepelova (2025)
Code	
Analysis	Shepelova (2025)
Material	
Instruments	Shepelova (2025)
Study/Analysis preregistration	
Study was not preregistered	—
Other	
Dictionary	Shepelova (2025)

## Data Availability

The analysis was conducted using SPSS Statistics 27.0.1. package and can be found on the Open Science Framework at Shepelova (2025).

## References

[r1] Anglim, J., & Horwood, S. (2021). Effect of the COVID-19 pandemic and Big Five personality on subjective and psychological well-being. Social Psychological & Personality Science, 12(8), 1527–1537. 10.1177/1948550620983047

[r2] Anglim, J., Horwood, S., Smillie, L. D., Marrero, R. J., & Wood, J. K. (2020). Predicting psychological and subjective well-being from personality: A meta-analysis. Psychological Bulletin, 146(4), 279–323. 10.1037/bul0000226 31944795

[r3] Ariza Toledano, D., & Ruiz-Olivares, R. (2023). Prosocial personality as a predictor of burnout in Spanish social workers. British Journal of Social Work, 53(1), 368–385. 10.1093/bjsw/bcac134 39514637

[r4] Ashton, M. C., & Lee, K. (2007). Empirical, theoretical, and practical advantages of the HEXACO model of personality structure. Personality and Social Psychology Review : An Official Journal of the Society for Personality and Social Psychologyf, 11(2), 150–166. 10.1177/1088868306294907 18453460

[r5] Barko, V. I., & Ostapovych, V. P. (2019). Україномовна адаптація опитувальника Баднера для використання в Національній поліції [Ukrainian-language adaptation of the Budner questionnaire for use in the National Police]. Psychological Journal, 5(8), 249–263. 10.31108/1.2019.5.8.16

[r6] Budner, S. (1962). Intolerance of ambiguity as a personality variable. Journal of Personality, 30(1), 29–50. 10.1111/j.1467-6494.1962.tb02303.x 13874381

[r7] Buecker, S., Luhmann, M., Haehner, P., Bühler, J. L., Dapp, L. C., Luciano, E. C., & Orth, U. (2023). The development of subjective well-being across the life span: A meta-analytic review of longitudinal studies. Psychological Bulletin, 149(7–8), 418–446. 10.1037/bul0000401

[r8] Busseri, M. A., & Erb, E. M. (2024). The happy personality revisited: Re-examining associations between Big Five personality traits and subjective well-being using meta-analytic structural equation modeling. Journal of Personality, 92(4), 968–984. 10.1111/jopy.12862 37462061

[r9] Carleton, R. N., Norton, M. P., & Asmundson, G. J. (2007). Fearing the unknown: A short version of the Intolerance of Uncertainty Scale. Journal of Anxiety Disorders, 21(1), 105–117. 10.1016/j.janxdis.2006.03.014 16647833

[r10] Carlson, J. F. (2024). Tolerance of ambiguity: Counterpoint to intolerance of uncertainty. Clinical Psychology, 31(2), 205–207. 10.1037/cps0000216

[r11] Cohen, S., & Wills, T. A. (1985). Stress, social support, and the buffering hypothesis. Psychological Bulletin, 98(2), 310–357. 10.1037/0033-2909.98.2.310 3901065

[r12] DeNeve, K. M., & Cooper, H. (1998). The happy personality: A meta-analysis of 137 personality traits and subjective well-being. Psychological Bulletin, 124(2), 197–229. 10.1037/0033-2909.124.2.197 9747186

[r13] DeYoung, C. G., Quilty, L. C., & Peterson, J. B. (2007). Between facets and domains: Ten aspects of the Big Five. Journal of Personality and Social Psychology, 93(5), 880–896. 10.1037/0022-3514.93.5.880 17983306

[r14] Diener, E., & Lucas, R. E. (2000). Personality and subjective well-being. In E. Diener & S. Suh (Eds.), *Subjective well-being: Scientific perspectives on enjoying life and having a good life* (pp. 213–229). MIT Press.

[r15] Diener, E., & Lucas, R. E. (2008). Personality and subjective well-being. In O. P. John, R. W. Robins, & L. A. Pervin (Eds.), *Handbook of personality: Theory and research* (3^rd^ ed., pp. 795–814). Guilford Press.

[r16] Diener, E., Oishi, S., & Lucas, R. E. (2003). Personality, culture, and subjective well-being: Emotional and cognitive evaluations of life. Annual Review of Psychology, 54(1), 403–425. 10.1146/annurev.psych.54.101601.145056 12172000

[r17] Fiori, M., Fischer, S., & Barabasch, A. (2022). Creativity is associated with higher well-being and more positive COVID-19 experience. Personality and Individual Differences, 194, 111646. 10.1016/j.paid.2022.111646 35400778 PMC8983605

[r18] Graziano, W. G., Habashi, M. M., Sheese, B. E., & Tobin, R. M. (2007). Agreeableness, empathy, and helping: A person × situation perspective. Journal of Personality and Social Psychology, 93(4), 583–599. 10.1037/0022-3514.93.4.583 17892333

[r19] Hayes, A. F. (2022). *Introduction to mediation, moderation, and conditional process analysis: A regression-based approach* (3^rd^ ed.). Guilford Press.

[r20] Hillen, M. A., Gutheil, C. M., Strout, T. D., Smets, E. M. A., & Han, P. K. J. (2017). Tolerance of uncertainty: Conceptual analysis, integrative model, and implications for healthcare. Social Science & Medicine, 180, 62–75. 10.1016/j.socscimed.2017.03.024 28324792

[r21] Himayeva, Y. A., & Malofeikina, K. O. (2019). Особистісна зрілість та суб’єктивне благополуччя в студентів денної та заочної форм навчання в університеті [Personality maturity and subjective well-being in students of full-time and correspondence forms of studying in the university]. Visnyk of V. N. Karazin Kharkiv National University: Series Psychology, 67, 15–24. 10.26565/2225-7756-2019-67-02

[r22] Hudek-Knežević, J., & Kardum, I. (2009). Five-factor personality dimensions and three health-related personality constructs as predictors of health. Croatian Medical Journal, 50(4), 394–402. 10.3325/cmj.2009.50.394 19673040 PMC2728392

[r23] Jiao, L., Jiang, W., Guo, Z., Xiao, Y., Yu, M., Xu, Y. (2023). Good personality and subjective well-being during the COVID-19 pandemic: A three-wave longitudinal study. Journal of Happiness Studies, 24, 589–606. 10.1007/s10902-022-00610-6 36568473 PMC9761042

[r24] Kashdan, T. B., & Steger, M. F. (2007). Curiosity and pathways to well-being and meaning in life: Traits, states, and everyday behaviors. Motivation and Emotion, 31(3), 159–173. 10.1007/s11031-007-9068-7

[r25] Klimanska, M. B., & Haletska, I. I. (2019). Українська адаптація короткого п’ятифакторного опитувальника особистості TIPI (TIPI-UKR) [Ukrainian adaptation of the Ten Item Personality Inventory (TIPI-UKR)]. Psychological Journal, 5(9), 57–74. 10.31108/1.2019.5.9.4

[r26] Kryazh, I. V., & Levenets, N. V. (2023). Суб’єктивне благополуччя людей з різним профілем відкритості особистості [Subjective well-being of people with different profiles of personality openness]. Visnyk of V. N. Karazin Kharkiv National University: Series Psychology, 75, 16–22. 10.26565/2225-7756-2023-75-02

[r27] Lucas, R. E., & Diener, E. (2008). Personality and subjective well-being. In O. P. John, R. W. Robins & L. A. Pervin (Eds.), *Handbook of personality: Theory and research* (3^rd^ ed., pp. 795–814). Guilford Press.

[r28] Margolis, S., & Lyubomirsky, S. (2018). The paradoxical effects of pursuing positive emotion: When and why wanting to feel happy backfires. In E. Diener, S. Oishi & L. Tay (Eds.), *Handbook of well-being* (pp. 1–17). DEF Publishers. https://digitalcommons.unomaha.edu/psychfacbooks/9/

[r29] Perrudet-Badoux, A., Mendelsohn, G., & Chiche, J. (1988). Development and validation of a scale for the subjective assessment of well-being. Journal of Anthropology and Human Biometry, 6(3–4), 121–134.

[r30] Purvanova, R. K., & Muros, J. P. (2010). Gender differences in burnout: A meta-analysis. Journal of Vocational Behavior, 77(2), 168–185. 10.1016/j.jvb.2010.04.006

[r31] Rammstedt, B., & John, O. P. (2007). Measuring personality in one minute or less: A 10‐item short version of the Big Five Inventory in English and German. Journal of Research in Personality, 41(1), 203–212. 10.1016/j.jrp.2006.02.001

[r32] Robins, R. W., Caspi, A., & Moffitt, T. E. (2002). It’s not just who you’re with, it’s who you are: Personality and relationship experiences across multiple relationships. Journal of Personality, 70(6), 925–964. 10.1111/1467-6494.05028 12498360

[r33] Romero, E., Villar, P., Luengo, M. Á., & Gómez-Fraguela, J. A. (2009). Traits, personal strivings and well-being. Journal of Research in Personality, 43(4), 535–546. 10.1016/j.jrp.2009.03.006

[r34] Serrano, C., Andreu, Y., & Murgui, S. (2020). The Big Five and subjective well-being: The mediating role of optimism. Psicothema, 32(3) 352–358. 10.7334/psicothema2019.392 32711670

[r35] Shepelova, M. V. (2025). *Materials* [OSF project page containing code, codebook, data, supplementary materials]. Open Science Framework. 10.17605/OSF.IO/GF8Q6

[r36] Singhal, A., Nema, P., Chakraborty, A., & Kumar, P. (2021). Big Five personality traits and well-being: Evidence from university students of India. Journal of Contemporary Issues in Business and Government, 27(1), 4064–4072. https://cibgp.com/index.php/1323-6903/article/view/844

[r37] Soto, C. J. (2015). Is happiness good for your personality? Concurrent and prospective relations of the Big Five with subjective well being. Journal of Personality, 83(1), 45–55. 10.1111/jopy.12081 24299053

[r38] Steel, P., Schmidt, J., & Shultz, J. (2008). Refining the relationship between personality and subjective well-being. Psychological Bulletin, 134(1), 138–161. 10.1037/0033-2909.134.1.138 18193998

[r39] Strohmaier, S., Pillai, M., Weitzer, J., Han, E., Zenk, L., Birmann, B. M., Bertau, M., Caniglia, G., Laubichler, M. D., Steiner, G., & Schernhammer, E. S. (2024). The relationship between Big Five personality traits and depression in the German-speaking D-A-CH region: An investigation of moderators and mediators. European Journal of Investigation in Health, Psychology and Education, 14(8), 2157–2174. 10.3390/ejihpe14080144 39194938 PMC11353565

[r40] Tanksale, D. (2015). Big Five personality traits: Are they really important for the subjective well-being of Indians? International Journal of Psychology : Journal International de Psychologie, 50(1), 64–69. 10.1002/ijop.12060 25611929

[r41] Taylor, S. E. (2011). Social support: A review. In H. S. Friedman (Ed.), *Oxford handbook of health psychology* (pp. 189–214). Oxford University Press. 10.1093/oxfordhb/9780195342819.013.0009

[r42] Turiano, N. A., Spiro, A., III, & Mroczek, D. K. (2012). Openness to experience and mortality in men: Analysis of trait and facets. Journal of Aging and Health, 24(4), 654–672. 10.1177/0898264311431303 22219209 PMC3649068

[r43] Wagaman, M. A., Geiger, J. M., Shockley, C., & Segal, E. A. (2015). The role of empathy in burnout, compassion satisfaction, and secondary traumatic stress among social workers. Social Work, 60(3), 201–209. 10.1093/sw/swv014 26173361

[r44] Wei, R., Kang, Y., & Wang, S. (2022). The relationship between tolerance of ambiguity and multilingualism revisited. System, 107, 102798. 10.1016/j.system.2022.102798

[r45] Wilt, J., & Revelle, W. (2009). Extraversion. In M. R. Leary & R. H. Hoyle (Eds.), *Handbook of individual differences in social behavior* (pp. 27–45). Guilford Press.

[r46] Winzer, R., Vaez, M., Lindberg, L., & Sorjonen, K. (2021). Exploring associations between subjective well-being and personality over a time span of 15–18 months: A cohort study of adolescents in Sweden. BMC Psychology, 9, 173. 10.1186/s40359-021-00673-9 34740376 PMC8569843

[r47] Xu, H., & Tracey, T. J. G. (2014). The role of ambiguity tolerance in career decision making. Journal of Vocational Behavior, 85(1), 18–26. 10.1016/j.jvb.2014.04.001

[r48] Xue, Y. (2024). Mediating effect of ambiguity tolerance in automated writing evaluation research model. American Journal of Social Sciences and Humanities, 9(2), 49–77. 10.55284/ajssh.v9i2.1178

[r49] Youfi, Q., & Brigui, H. (2024). The role of uncertainty tolerance in enhancing intercultural communication competence among high school students in Morocco: A quantitative analysis. International Journal of Linguistics, Literature and Translation, 7(9), 152–159. 10.32996/ijllt.2024.7.9.18

[r50] Zavhorodnia, O. V., Shepelova, M. V., & Hulko, Yu. A. (2025). Динаміка суб’єктивного благополуччя молоді: Від довоєнного періоду до сьогодення [Dynamics of subjective well-being of youth: From the pre-war period to the present]. Габітус, 70, 77–82. 10.32782/2663-5208.2025.70.12

[r51] Zuo, T. (2024). Tolerance for ambiguity and happiness: Mediating role of creativity among Chinese college students across majors. Current Psychology, 43, 27690–27713. 10.1007/s12144-024-06396-5

[r52] Zuo, T. (2025). From tolerance for ambiguity to stress and anxiety: The mediating role of need for cognitive closure among Chinese university students. Psychological Reports, 128(6), 3967–3998. 10.1177/00332941231212833 37936412

